# Luteolin delays the progression of IgA nephropathy by attenuating inflammation, oxidative stress and reducing extracellular matrix accumulation through activating the Nrf-2/HO-1 pathway

**DOI:** 10.3389/fphar.2025.1530655

**Published:** 2025-07-08

**Authors:** Dong-Yu Liang, Shao-Hua Cong, Lin-Hui Li, Qing-Qing Yi, Li-Ping Tang, Li-Ou Cao

**Affiliations:** ^1^ Clinical Research Center, Jiading District Central Hospital Affiliated Shanghai University of Medicine and Health Sciences, Shanghai, China; ^2^ Department of Stomatology, Jiading District Central Hospital Affiliated Shanghai University of Medicine and Health Sciences, Shanghai, China; ^3^ Department of Scientific Research, Jiading District Central Hospital Affiliated Shanghai University of Medicine and Health Sciences, Shanghai, China; ^4^ Department of Nephrology, Molecular Cell Lab for Kidney Disease, Ren Ji Hospital, Shanghai Jiao Tong University School of Medicine, Shanghai, China

**Keywords:** IgA nephropathy, ROS, extracellular matrix, Nrf-2, luteolin (LUT)

## Abstract

IgA nephropathy (IgAN) is the most common primary glomerulonephritis and the main cause of end-stage renal disease (ESRD). Luteolin (Lut), which is present in various plants, has anti-inflammatory and antioxidant properties under numerous medical conditions. This study aimed to investigate the therapeutic effects and potential mechanisms of Lut on IgAN. Mouse models of IgAN and HBZY-1 cells stimulated with Gd-IgA1 were used as experimental objects. Renal pathology, inflammation, reactive oxygen species (ROS) levels, and extracellular matrix (ECM) accumulation were measured. The results indicated that Lut improved renal pathological damage, reduced the levels of inflammatory cytokines, decreased ROS levels, and attenuated ECM accumulation. Moreover, Lut promoted the activation of the Nrf-2/HO-1 pathway. Furthermore, blocking Nrf2 reversed the suppressive effects of Lut on inflammation, oxidative stress, and the expression of ECM proteins in mesangial cells stimulated with Gd-IgA1. In conclusion, the protective effect of Lut against IgAN may occur by triggering the Nrf2/HO-1 pathway, thereby suppressing inflammation, oxidative stress, and ECM deposition.

## Introduction

IgA nephropathy (IgAN) is a common glomerular disease worldwide. The prevalence of IgAN varies greatly across continents, countries, and regions within countries. The incidence of IgAN in China accounts for 26%–34% of primary glomerular diseases. In IgAN, immune complexes mainly comprising IgA1 accumulate in the mesangial region of the glomerulus. The main clinical manifestations of IgAN are recurrent gross hematuria or microscopic hematuria, which may be accompanied by varying degrees of proteinuria. Studies have shown that IgAN is one of the main causes of end-stage renal disease (Stamellou et al., 2024). Therefore, further study of the pathogenesis of IgAN is necessary. Previous studies have shown a close correlation between the oxidative stress response and IgAN-induced kidney injury (Li et al., 2024), which is highly important for exploring new therapeutic targets. Moreover, studies have shown that the Nrf2/HO-1 signaling pathway plays an important role in regulating oxidative stress and treating intestinal and kidney diseases (Liu et al., 2024a; Wang et al., 2024). However, the mechanism by which this pathway acts in IgAN remains unclear.

Nuclear factor erythroid-derived 2-related factor 2 (Nrf2) is a stimulus-inducible transcription factor that is directly downstream of reactive oxygen species (ROS) production. Under oxidative stress conditions, Nrf2 is transported to the nucleus, where it activates antioxidant response element (ARE)-dependent gene transcription, clears ROS, and maintains cellular redox stability (Jo et al., 2024). Heme oxygenase-1 (HO-1) is a key antioxidant enzyme regulated by Nrf2. Many studies have confirmed that Nrf2 expression upregulation has a protective effect in IgAN animal models (Wu et al., 2023; Yang et al., 2023; Yang et al., 2013). Therefore, activating the Nrf2/HO-1 pathway is a meaningful therapeutic approach for protecting against IgAN. A recent study revealed that Nrf2 expression upregulation could inhibit the occurrence of oxidative stress disease related to diabetes; however, the specific mechanism requires further exploration (Qiu et al., 2023).

In traditional Chinese medicine, patients with IgAN are mainly characterized by blood stasis, which is often accompanied by hematuria. Hemostatic drugs alone can stop bleeding, but not eliminate it; blood-activating drugs alone can eliminate blood stasis, but not stop it. Luteolin, a natural antioxidant, is widely present in many plants, such as *Chrysanthemum indicum* L. Owing to its dual effects of promoting blood circulation and stopping bleeding, it is an important drug in the clinical treatment of patients with blood stasis syndrome (Liu et al., 2024b). Luteolin has many beneficial functions, such as anti-inflammatory, antioxidative, antitumor, and antiviral effects (Zhu et al., 2024). Increasing evidence has demonstrated that luteolin can reduce the secretion of proinflammatory factors such as tumor necrosis factor α (TNF-α) and interleukin 6 (IL-6), thus inhibiting the inflammatory response (Peng et al., 2024). Luteolin has been used to treat inflammation-related diseases, including multiple sclerosis and autoimmune thyroiditis (Contarini et al., 2019; Xia et al., 2016). In addition, research by Vukelić et al. (2020) illustrated the anti-inflammatory properties of luteolin, modulating the ERK signaling pathway to regulate apoptosis and autophagy and ameliorating experimental colitis in murine models. However, the effect of luteolin on renal injury is poorly understood. Here, we report the suppressive effects of luteolin on inflammation, oxidative stress, and extracellular matrix (ECM) accumulation in Gd-IgA1-induced glomerular mesangial cells and a mouse IgAN model. Our results provide a new perspective on the mechanism of luteolin in treating IgAN.

## Materials and methods

### Animal experiment design

Twenty-six six-week-old SPF-grade BALB/c female mice were obtained from Shanghai Victoria Laboratory Animals (Shanghai, China) and bred under controlled environmental conditions. All experimental equipment and procedures were approved by the Animal Care and Use Committee of the Shanghai University of Medicine and Health Sciences on 23 March 2023 (approval number: RA-2023–019).

After 1 week of adaptation during which the mice where fed a balanced diet, the mice were randomly distributed into four groups: (1) control (n = 6); (2) luteolin (Lut) (n = 6); (3) IgAN (model group) (n = 7); and (4) IgAN + Lut (n = 7).

A mouse model of IgAN was successfully developed by administering bovine serum albumin (BSA) at a dosage of 800 mg/kg daily for a period of 9 weeks. Castor oil and carbohydrate chloride (CCl_4_) were mixed at a ratio of 5:1; of this mixture, 0.1 mL was injected subcutaneously every week, and 0.06 mL was injected intraperitoneally once for 2 weeks. At weeks 6 and 8, 50 μg of lipopolysaccharide (LPS) was administered *via* the tail vein. The normal control group received an equivalent dose of distilled water per mouse, and normal saline was administered through subcutaneous, intraperitoneal, or tail vein injection. The mice were provided food and water *ad libitum* during the experiment. In week 9, two mice were randomly selected from the model group, and the IgAN model was confirmed to be successfully established by glomerular IgA immunofluorescence. No mice died during model induction.

**FIGURE 1:**
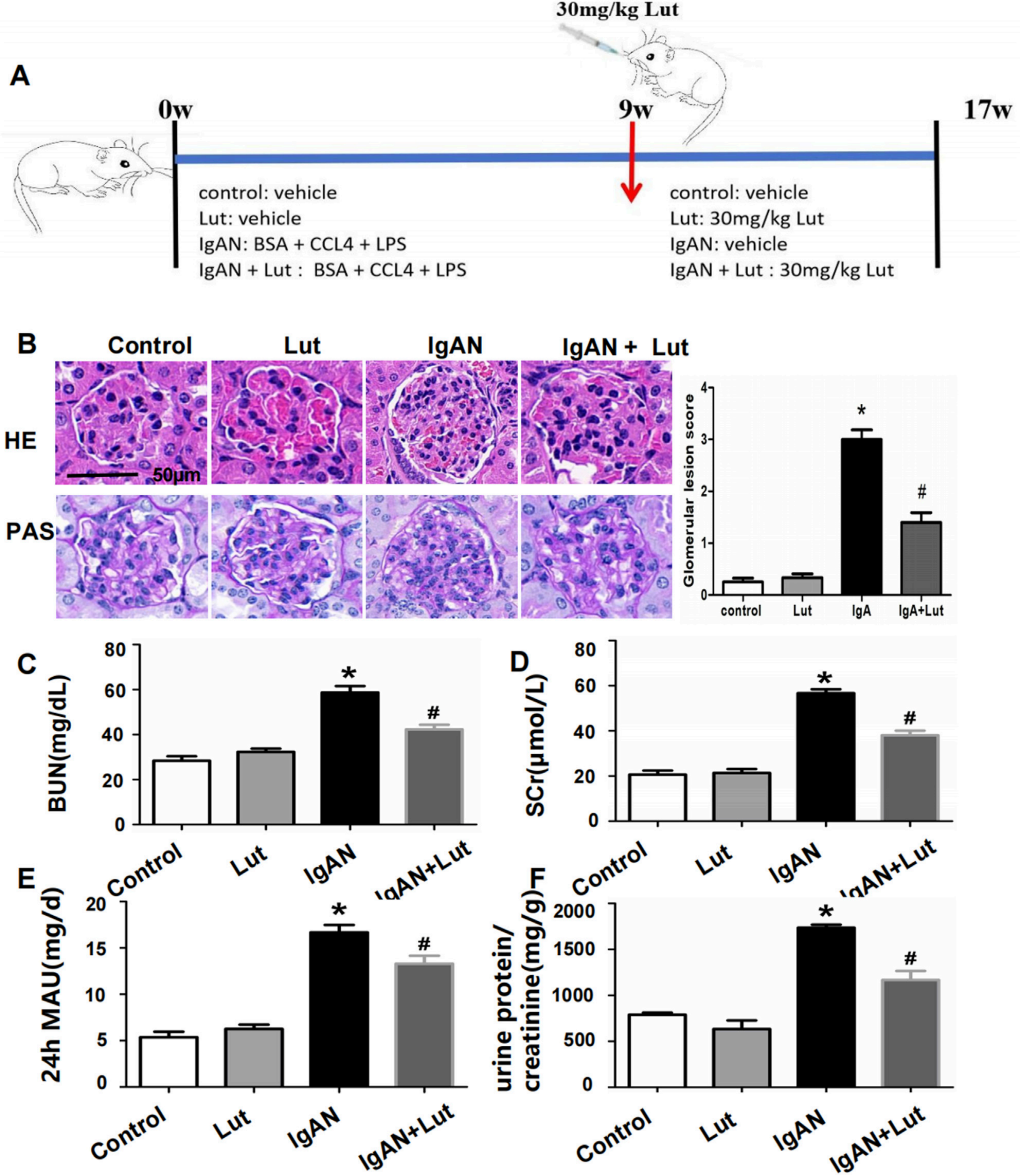
Effects of Lut on the physicochemical characteristics and renal morphological changes of IgAN mice**. (A)** A schematic workflow for the induction and treatment plan. **(B)** Histopathological changes in IgAN mice were observed via HE and PAS staining. **(C)** BUN levels. **(D)** Serum creatinine level. **(E)** Urine 24-hour MAU. **(F)** Urine protein/creatinine ratio. Results are presented as mean ± SEM (n = 6), *p < 0.05 vs control mice; #*p* < 0.05 vs IgAN mice.

Starting at week 10, each mouse in the Lut treatment group was given 30 mg/kg of Lut orally once a day for 8 weeks. A schematic of the induction and treatment design is shown in Figure 1A. Lut (purity ≥99%) was purchased from MCE (New Jersey, United States), dissolved in DMSO, and diluted in saline. The control group and IgAN model groups received an equivalent volume of distilled saline. The dose of Lut administered was selected based on previous studies and our preliminary experimental results (Boeing et al., 2020). This treatment continued until week 17. No mice died during the experiment. At its end, all mice were anesthetized with 5% isoflurane and euthanized *via* cervical dislocation.

### Biochemical assessment

Prior to euthanasia, blood samples were obtained and then centrifuged at 3,000 rpm for 10 min. The serum was collected for subsequent analysis. Blood urea nitrogen (BUN) and serum creatinine levels were determined with enzyme-linked immunosorbent assay (ELISA) kits (Jiancheng, China; C013-2-1 and C011-2-1). During the 16th week, the mice were placed in metabolic cages for 24 h, and their urine samples were collected. After the urine volume was recorded, it was mixed well, and 5 mL was collected in a centrifuge tube for the analysis of urinary albumin and urinary creatinine with ELISA kits (Jiancheng, Nanjing, China, A028-2-1 and C011-2-1), after which the urinary albumin/creatinine ratio (ACR) was calculated.

### Histological evaluation and immunohistochemistry

After being fixed with 4% paraformaldehyde (PFA) and embedded in paraffin, the renal tissues were cut into 4-μm-thick sections. The sections were stained with hematoxylin–eosin (HE), Periodic Acid-Schiff (PAS), and Masson. Light microscopy was subsequently used to observe pathological changes in the renal tissue. Histological analysis was performed by two independent investigators in a blinded manner. Briefly, inflammation and fibrosis in the glomerular compartments were classified into four grades, from normal to severe, and expressed as 0–3 to indicate glomerular interaction scores for each group. For each mouse, approximately 20 glomeruli were observed under a 200× microscope to examine pathological changes, including mesangial cell and mesangial matrix proliferation, mesangial matrix expansion, glomerular sclerosis, and fibrotic changes.

The paraffin-embedded kidney samples were rehydrated, subjected to antigen repair via citric acid and serum blockade, and then incubated with primary antibodies (anti-TNF-ɑ: Abcam, ab307164, 1:1,000; anti-IL-1β: Abcam, ab283818, 1:500; anti-IL-6: Abcam, ab9324, 1:500) overnight at 4°C. Peroxidase-coupled secondary antibodies were introduced, and the samples were incubated for half an hour. Following incubation with diaminobenzidine, the sections were observed under a light microscope.

### Evaluation of ROS, MDA, and GSH levels

Following washing with normal saline, the kidney tissues were homogenized in radioimmunoprecipitation assay lysis buffer with phenylmethylsulfonyl fluoride and phosphatase inhibitors via a homogenizer, and the collected cells were subjected to ultrasound fragmentation for analysis. After centrifugation for 10 min, the supernatant was collected, and ROS levels were measured using an ROS detection kit (Beyotime, Shanghai, China); malondialdehyde (MDA) and glutathione (GSH) (Jiancheng Bioengineering Institute, Nanjing, China) test kits were also applied. Absorbances were measured at 570 nm, 532 nm, and 405 nm with a spectrophotometer (Thermo Fisher Scientific, MA, United States).

### Cell culture

Rat glomerular mesangial cells (HBZY-1) were acquired from the BeNa Culture Collection (BNCC, Beijing, China). The cells were cultured in Dulbecco’s modified Eagle’s medium (DMEM) supplemented with 10% fetal bovine serum (FBS), 100 μg/mL streptomycin, and 100 U/mL penicillin at 37°C in a 5% CO2 humidified atmosphere.

### Cell viability assay

The impact of Lut on HBZY-1 cells was investigated in the presence of PBS or Gd-IgA1 stimulation via CCK-8 analysis. Initially, HBZY-1 cells were plated in 96-well plates at a density of 2 × 10^3^ cells/well and incubated for 24 h. Next, the cells were exposed to various concentrations of Lut (0, 1.5, 3, 6, 12, 25, or 50 μM) for an additional 24-hour period. After treatment, CCK-8 reagent (Dojindo) was added to the cells, and optical density (OD) was assessed via a microplate reader after a 1.5-hour incubation period. Cell viability was calculated as the percentage of the average absorbance relative to that of the control group. Cell viability (%) = (OD_treated group_/OD_Control group_) × 100%.

### Identification of inflammatory cytokines

IgAN progression is closely correlated with inflammation and oxidative stress. Hence, we investigated the impact of Lut on the inflammation and oxidative stress induced by Gd-IgA1 in HBZY-1 cells. HBZY-1 cells were cultured in six-well plates at a density of 4 × 10^5^ cells/well and treated with 0, 5, or 10 μM Lut and Gd-IgA1 (500 μg/mL) in the presence or absence of ML385 (an Nrf2 inhibitor) for 24 h. TNF-α, IL-6, and IL-1β levels were then measured using appropriate ELISA kits following the manufacturer’s instructions.

### ROS level evaluation

Intracellular ROS production was measured by DCFH–DA staining. Gd-IgA1-stimulated HBZY-1 cells were treated with different doses of Lut (0, 5, or 10 μM) in the presence or absence of ML385. After being cultured for 24 h, the cells were washed three times with PBS and then exposed to DCFH–DA (Sigma, St. Louis, MO, United States) in the dark at 37°C for 30 min. The cells in each experimental group were subsequently subjected to fluorescence microscopy analysis via excitation and emission wavelengths of 485 and 520 nm, respectively. In addition, ROS levels were evaluated using an ROS assay kit (Jiancheng Bioengineering Institute, China) according to the manufacturer’s instructions.

### Immunofluorescence assay

HBZY-1 cell slides subjected to various treatments were fixed with 4% paraformaldehyde for 20 min at room temperature and permeabilized with 0.1% Triton X-100 for 10 min. After blocking with a blocking solution comprising 5% of skim milk in 0.01 M PBS for 1 h, the cells were incubated at 4°C overnight with a primary antibody targeting TGF-β1 (ab308300, 1:250) and subsequently incubated for 1 h with a goat anti-rabbit secondary antibody (Abcam) at room temperature. The slides were stained with DAPI for 5 min to visualize the cell nuclei, and the images of the cells were examined via a laser confocal microscope (TCSSP2; Leica).

### Western blotting

The obtained renal tissues and cells were collected and subjected to lysis using RIPA lysis buffer (Beyotime, Shanghai). A bicinchoninic acid assay kit (Pierce Biotechnology) was then used to assess protein concentrations. Each sample containing an equal concentration of protein was subsequently separated via 12% SDS‒PAGE and transferred to PVDF membranes (Millipore, Billerica, China). Following blocking with 5% nonfat milk in TBST at room temperature for 1 h, primary antibodies against ASK1 (1:1,000, SAB4501866), fibronectin (1:1,000, ab2413), TGF-β1 (1 μg/mL, ab92486), collagen IV (1:1,000, ab6586), Nrf2 (1:1,000, ab137550), HO-1 (1:2000, ab13243), and NQO1 (1 μg/mL, ab34173) were incubated with the membranes at 4°C overnight. After washing with TBST three times, the secondary antibodies were added for 1 h at room temperature. The protein bands were visualized with an Odyssey^®^ CLx Infrared Imaging System and quantified with ImageJ software.

### RT–PCR

An RNAfast 200 kit (Fastagen, Shanghai, China) was used to extract cellular mRNA. A SureScript First-Strand cDNA Synthesis Kit (GeneCopoeia, United States) was used to reverse-transcribe the cDNA. Real-time PCR experiments were performed using SYBR Premix Ex Taq reagent (Takara) and an ABI 7500 system. The sequences used were as follows: homo GAPDH: forward 5′-GCA​CCG​TCA​AGG​CTG​AGA​AC -3′, reverse 5′-TGG​TGA​AGA​CGC​CAG​TGG​A -3′; FN: forward 5′-TAC​GCG​TCT​TGG​ACT​CTT​G-3′, reverse 5′- CGG​ACT​CCA​GGA​AGT​GCG​TC-3′. LN: forward 5′-GTT​GCC​GTT​CAC​GGT​TCC​TA-3′; reverse 5′- AGG​ATT​CGT​ACT​GTT​ACC​CGT​CCA -3′.

### Statistical analysis

All data were analyzed via SPSS 23.0 software. A Student’s t*-*test was performed for comparisons between two groups, and a one-way ANOVA was used to compare multiple groups. The sample size of the animal studies was six per time point per group, and the results are presented as the mean ± SEM. For the *in vitro* studies, the sample size for each group was three unless indicated otherwise, and the data are expressed as the means ± SD. With a two-tailed test, *p* < 0.05 was considered to indicate significance.

## Results

### Effects of Lut on physicochemical characteristics and renal morphological changes in IgAN mice

To evaluate the impact of Lut on kidney injury in IgAN mice, we measured their serum Scr, BUN, and urine 24-hour MAU levels. Compared with the control group, the IgAN group presented significant increases in the serum Scr, BUN, and 24-hour urine MAU levels. Lut intervention led to a noteworthy reduction in the levels of Scr, BUN, and urine 24-hour MAU in IgAN mice (Figures 1C–F). The effects of Lut on morphological changes in IgAN mice were then observed via HE and PAS staining (Figure 1B). HE staining revealed significant pathological changes, such as spheroid hypertrophy and mesangial matrix dilation, in the kidneys of IgAN mice. These damage changes dramatically improved after Lut treatment.

### Lut attenuates the levels of inflammatory cytokines and reduces oxidative stress in the kidneys of mice with IgAN

**FIGURE 2:**
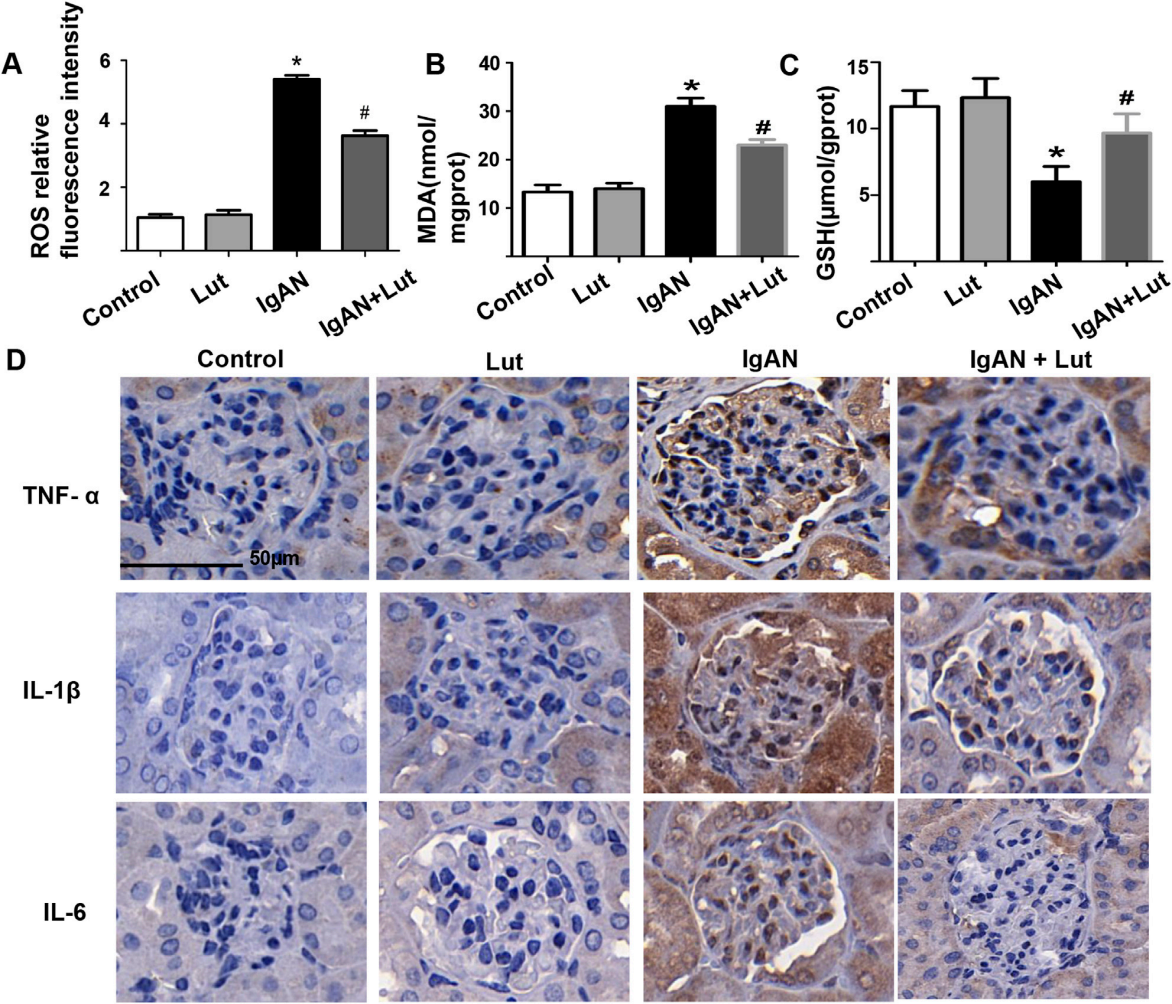
Lut ameliorates oxidative stress and inflammation in the kidneys of IgAN mice**. (A)** The fluorescence intensity of ROS was analyzed via ImageJ. **(B)** Levels of MDA. **(C)** Levels of GSH. **(D)** Protein expression levels of inflammatory cytokines TNF-ɑ, IL-1β, and IL-6 were detected by IHC staining. Results are presented as mean ± SEM (n = 6), *p < 0.05 vs control mice; #*p* < 0.05 vs IgAN mice.

As shown in Figure 2D, the secretion of TNF-α, IL-1, and IL-6 was markedly increased in IgAN mice, whereas Lut treatment significantly reduced the levels of these proinflammatory cytokines compared with those in the IgAN group. In addition, oxidative stress was detected. Compared with control mice, IgAN mice presented increased production of ROS, whereas Lut reduced the level of ROS in the IgAN group (Figure 2A). Moreover, GSH levels in the kidneys of IgAN mice were significantly reduced, whereas MDA levels were significantly increased. Lut treatment significantly inhibited the decrease in GSH levels and the increase in the MDA content (Figures 2B,C), indicating that Lut can alleviate oxidative stress-induced renal damage in IgAN mice.

### Lut ameliorates the deposition of ECM proteins in the kidneys of IgAN mice

**FIGURE 3:**
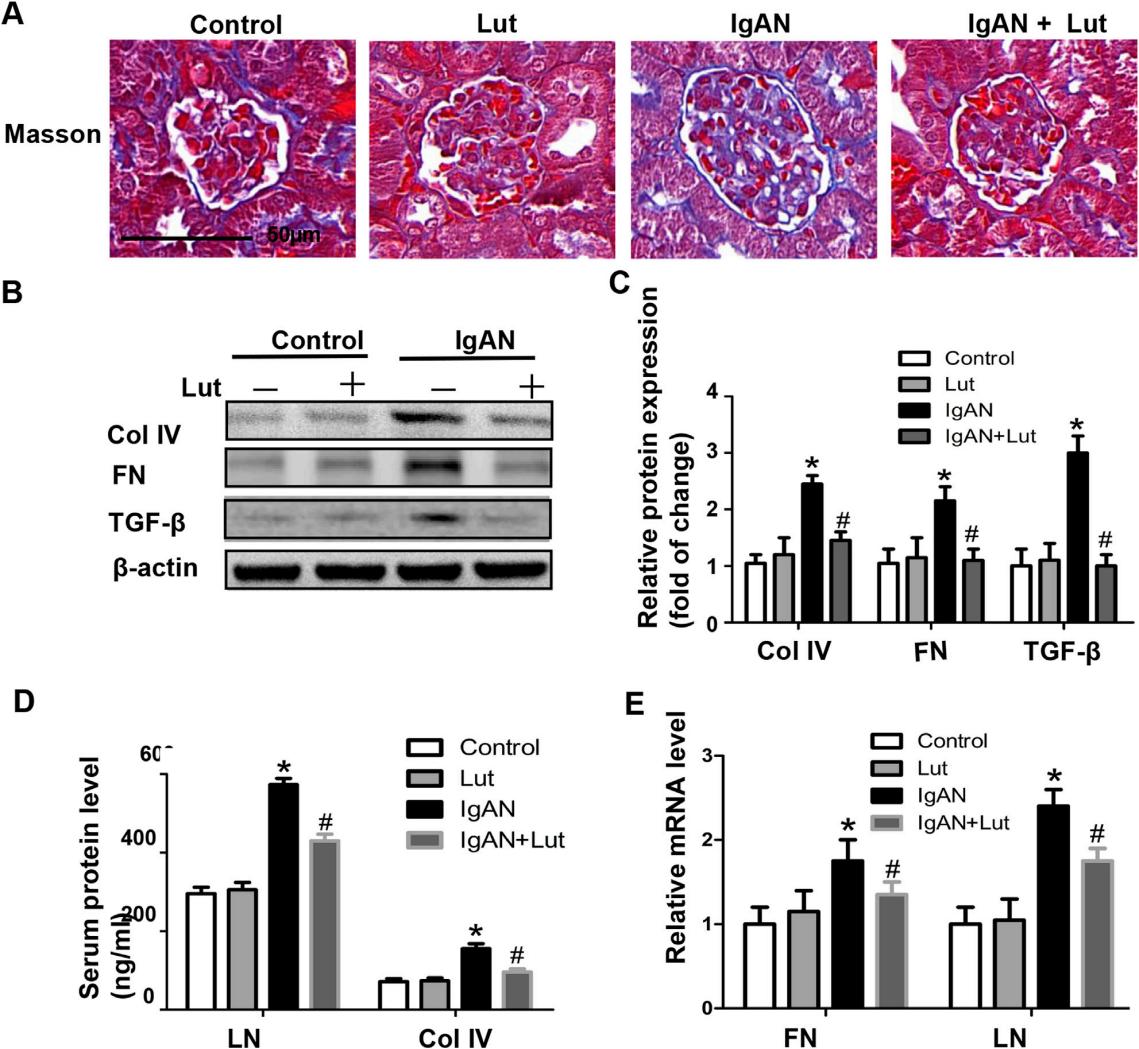
Lut reduced ECM accumulation in the kidneys of IgAN mice**. (A)** ECM accumulation in IgAN mice observed using Masson’s trichrome staining. **(B)** Renal Col IV, FN, and TGF-β protein levels assessed by Western blotting. **(C)** Quantification of Western blotting data. **(D)** Serum LN and Col IV protein levels detected via ELISA. **(E)** Relative mRNA expression of FN and LN detected by real-time PCR. Results presented as the mean ± SEM (n = 6), *p < 0.05 vs control mice; #*p* < 0.05 vs IgAN mice.

Prolonged inflammation and oxidative stress led to the expansion of the mesangial matrix, resulting in the progression of renal fibrosis in IgAN mice, as indicated by a significant increase in the area of fibrosis in their kidneys compared with that in control mice. These effects were mitigated by the administration of Lut (Figure 3A). Consistent with the histological assessment, the inhibition of excessive secretion of extracellular matrix proteins, including collagen 4 (Col IV) and fibronectin (FN), by Lut was validated by Western blotting (Figure 3B). Transforming growth factor-β (TGF-β) is a direct stimulator of these overexpressed ECM proteins. Hence, the expression of TGF-β was examined, revealing that Lut effectively reduced the protein expression level of TGF-β (Figures 3B,C). Furthermore, the serum protein and mRNA expression levels of biomarkers related to fibrosis, such as Col IV, FN, and laminin (LN), were also reduced in the Lut treatment group (Figures 3D,E). Overall, these findings indicate that Lut can alleviate the fibrotic process of IgAN nephropathy.

### Lut suppresses Gd-IgA1-induced HBZY-1 cell proliferation

**FIGURE 4:**
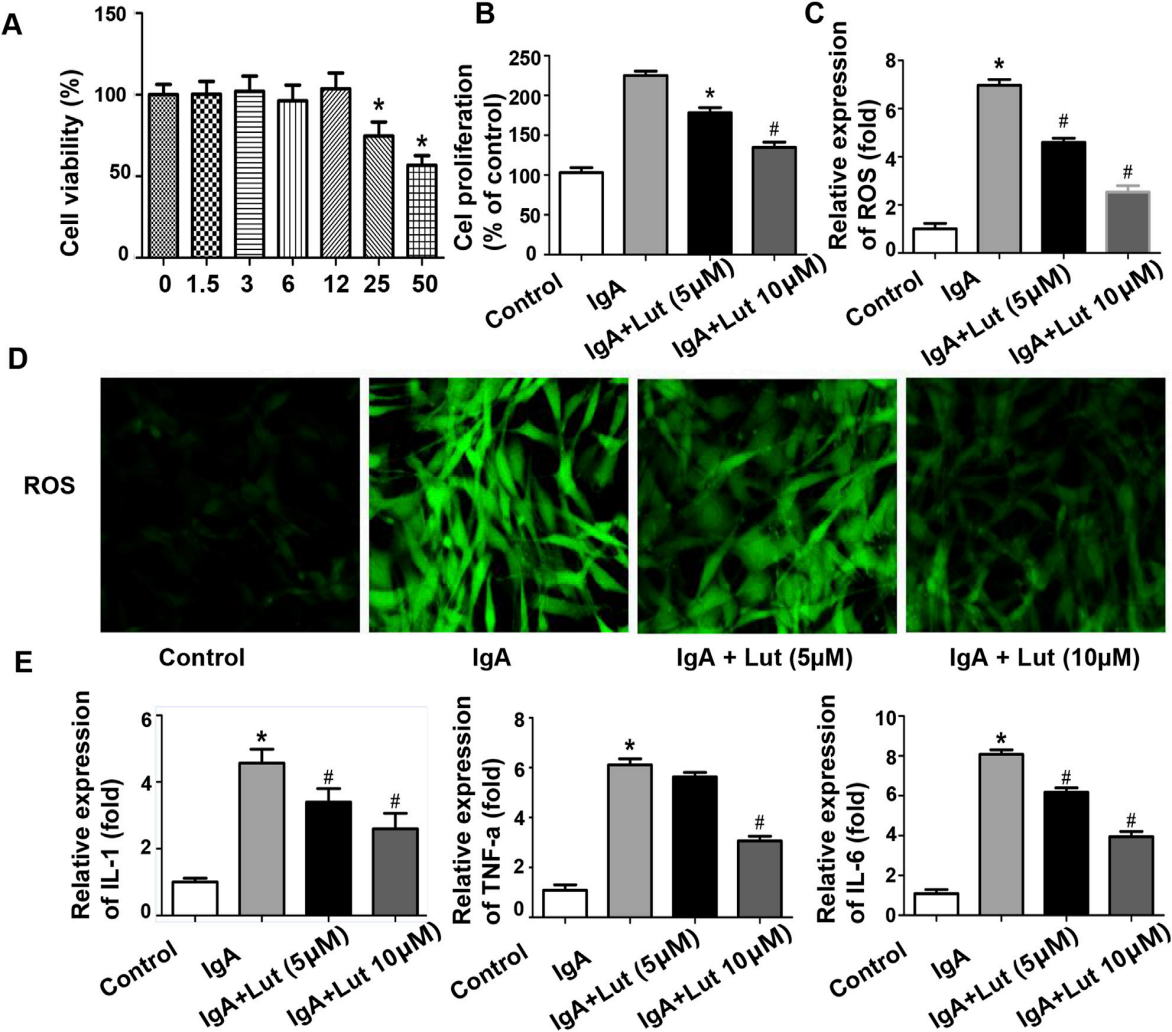
Effects of Lut on inflammation and oxidative stress induced by Gd-IgA1 in HBZY-1 cells. **(A)** Cell viability was determined after treatment with different concentrations of Lut. **(B)** Effects of Lut on Gd-IgA1-induced mesangial cell proliferation. **(C)** ROS levels quantified by ELISA. **(D)** ROS generation detected by DCFH-DA staining (100×). **(E)** Production of the inflammatory cytokines TNF-ɑ, IL-1β, and IL-6 was detected via ELISA. Results presented as means ± standard deviations (n = 3), *p < 0.05 vs the control group; #*p* < 0.05 vs the IgA group.

To investigate the impacts of Lut on normal glomerular mesangial cells, we evaluated cell proliferation via a CCK-8 assay (Figure 4A). The viability of the HBZY-1 cells did not significantly change following exposure to various concentrations of Lut (0, 1.5, 3, 6, or 12 μM). Thus, 5 and 10 μM Lut were used in the subsequent experiments. Next, the impact of Lut on the proliferation of Gd-IgA1-induced glomerular mesangial cells was examined. Compared with the control, Gd-IgA1 significantly stimulated cell growth. However, Lut inhibited cell proliferation in a dose-dependent manner (Figure 4B).

### Effects of Lut on inflammatory cytokines and oxidative stress induced by Gd-IgA1 in HBZY-1 cells

As shown in Figure 4E, a significant increase in TNF-α, IL-1, and IL-6 secretion was observed following Gd-IgA1 induction, whereas Lut treatment effectively reduced the levels of these proinflammatory cytokines compared with those in the IgA group. Furthermore, ROS levels were assessed to determine the degree of oxidative stress. Compared with no treatment, Gd-IgA1 administration led to an increase in ROS production, whereas Lut mitigated the increase in ROS levels in the Gd-IgA1-induced cells (Figures 4C,D).

### Effects of Lut on the deposition of ECM proteins induced by Gd-IgA1 in HBZY-1 cells

**FIGURE 5:**
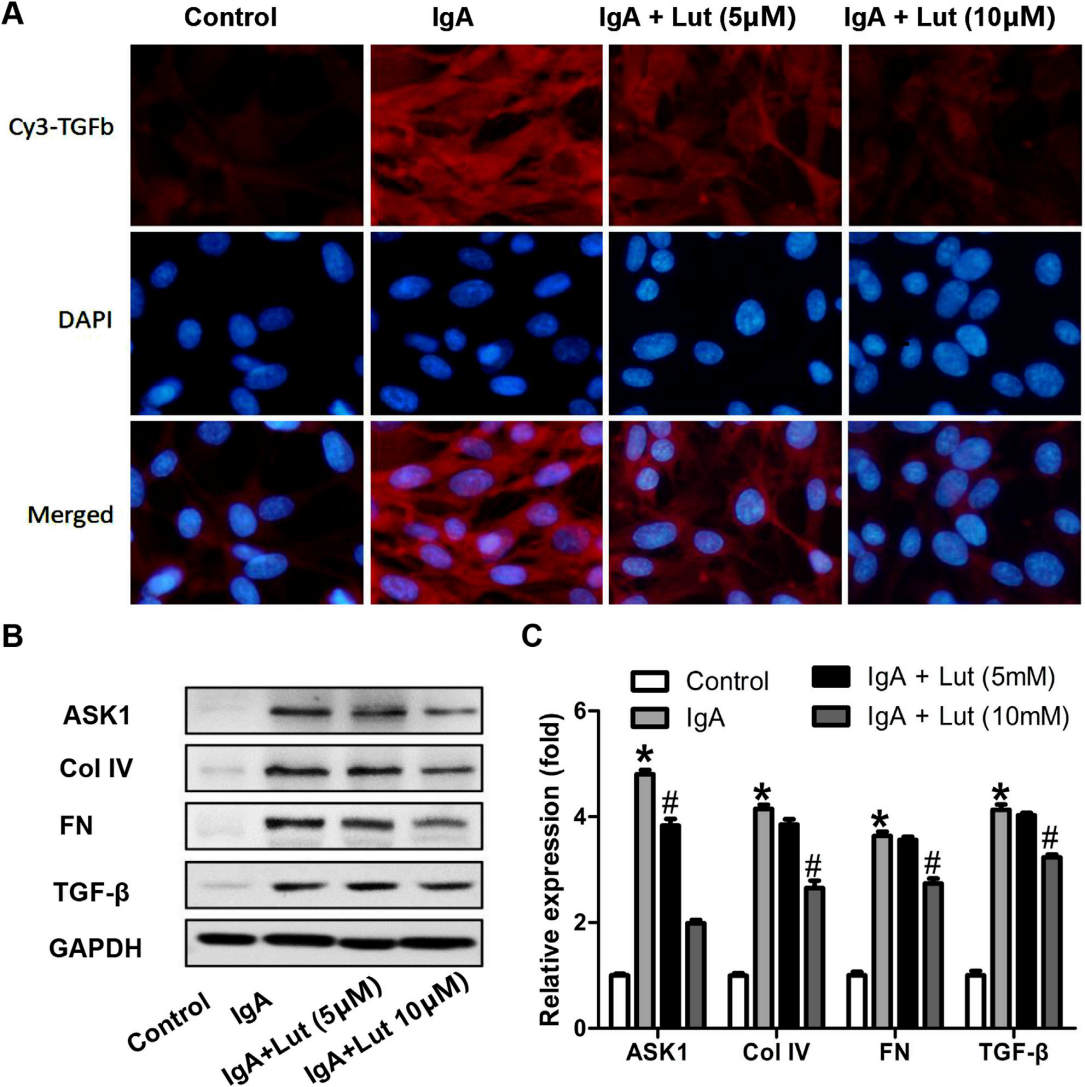
Effect of Lut on deposition of ECM proteins under Gd-IgA1 stimulation. (**A)** TGF-β protein levels in the different groups were detected via immunofluorescence assay at 200×. **(B)** ASK1, Col IV, FN, and TGF-β levels were evaluated by Western blotting. **(C)** Quantification of Western blotting data. Results presented as the means ± standard deviations (n = 3), *p < 0.05 vs the control group; #*p* < 0.05 vs the IgAN group.

Proteins associated with ECM accumulation were assessed to investigate whether Lut affects Gd-IgA1-induced ECM accumulation in HBZY-1 cells. Following exposure to Gd-IgA1, an increase in TGF-β1 fluorescence intensity was observed via an immunofluorescence assay. In contrast, Lut treatment significantly reduced the fluorescence intensity compared with that in cells induced by Gd-IgA1 (Figure 5A). Moreover, the protein expression of ASK1, FN, TGF-β1, and Col IV in HBZY-1 cells was strongly induced by Gd-IgA1. Nevertheless, the regulation of the four proteins upregulated by Gd-IgA1 was inhibited by Lut (Figures 5B,C). This finding highlights the potential inhibitory effect of Lut on ECM accumulation.

### Lut activated the Nrf2/HO-1 pathway in the IgAN mouse model and Gd-IgA1-stimulated HBZY-1 cells

**FIGURE 6:**
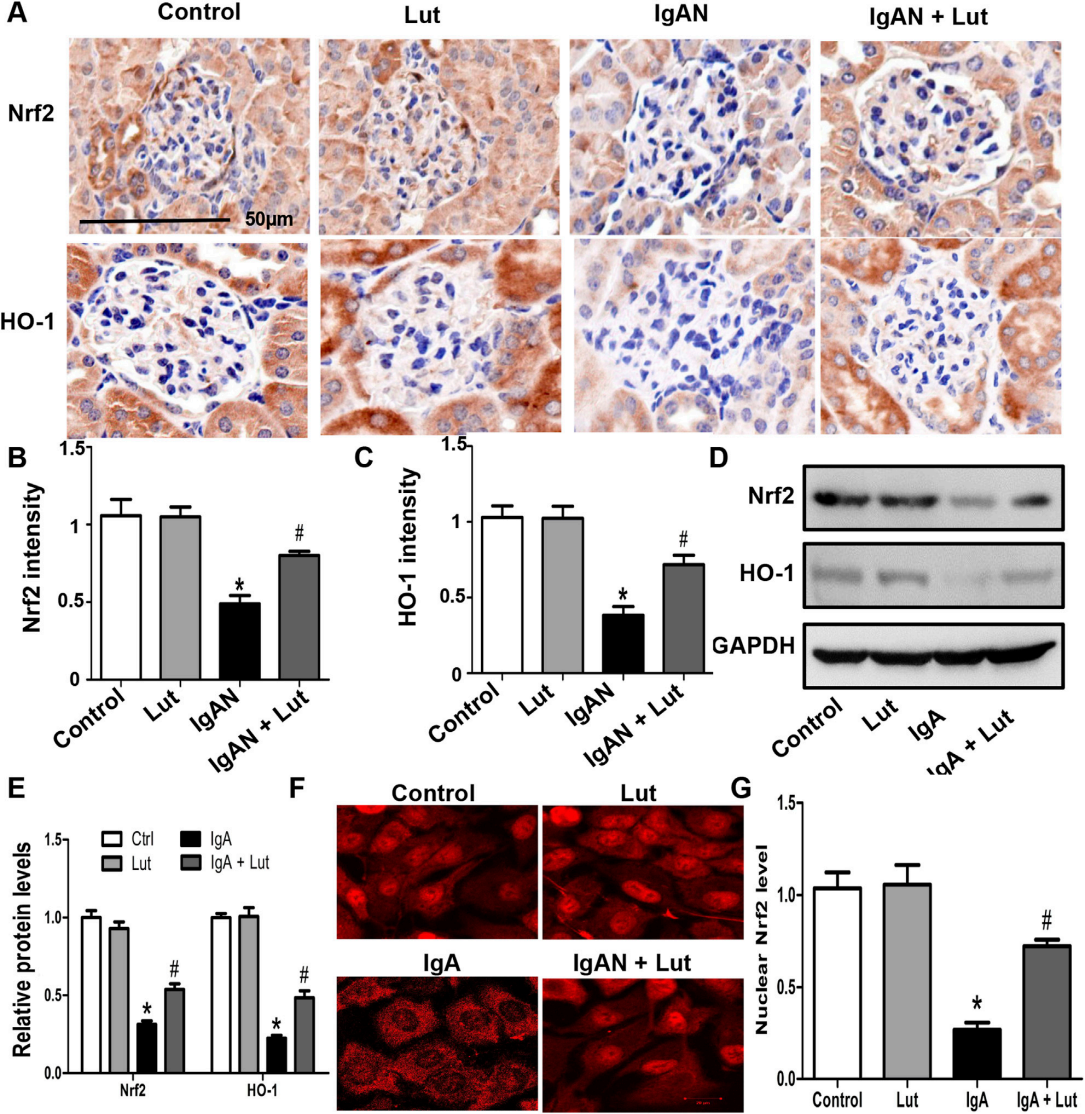
Lut activated the Nrf2/HO-1 pathway in the IgAN mouse model and Gd-IgA1-stimulated HBZY-1 cells. **(A)** Renal Nrf2 and HO-1 protein expression levels detected by IHC staining **(B, C).** Quantification of IHC staining. **(D)** Protein expression of Nrf2 and HO-1 in HBZY-1 cells subjected to different treatments was determined by Western blotting. **(E)** Quantification of Western blotting data. **(F)** The expression and intracellular localization of Nrf2 in HBZY-1 cells subjected to different treatments were detected via confocal microscopy. **(G)** Quantification of confocal images. Results presented as means ± standard deviations (n = 3), *p < 0.05 vs the control group; #*p* < 0.05 vs the IgAN group.

Considering the pivotal role of Nrf2 in oxidative stress and ECM accumulation, we examined the impact of Lut on the Nrf2/HO-1 pathway in the IgAN mouse model. IHC staining revealed decreases in Nrf2 and HO-1 protein levels in the kidneys of IgAN mice compared with those in the kidneys of control mice. Conversely, there was a significant increase in Nrf2 and HO-1 protein levels in the microsections of IgAN mice that were treated with Lut (Figures 6A–C).

Moreover, *in vitro* Western blotting further revealed a decrease in Nrf2 and HO-1 protein levels in HBZY-1 cells exposed to Gd-IgA1, with the subsequent reversal of these alterations upon Lut treatment (Figures 6D,E). An investigation was subsequently conducted to explore whether the promotion of Nrf2 nuclear translocation by Lut could be observed through immunofluorescence chemical staining. Confocal microscopy analysis revealed a notable decrease in the nuclear translocation of Nrf2 in the HBZY-1 cells exposed to Gd-IgA1 compared with the control cells. Upon exposure to Gd-IgA1, there was a notable increase in the nuclear translocation of Nrf2 in HBZY-1 cells following Lut treatment (Figures 6F,G).

### Nrf2 inhibition exacerbates Gd-IgA1-induced inflammation, oxidative stress, and ECM accumulation

**FIGURE 7:**
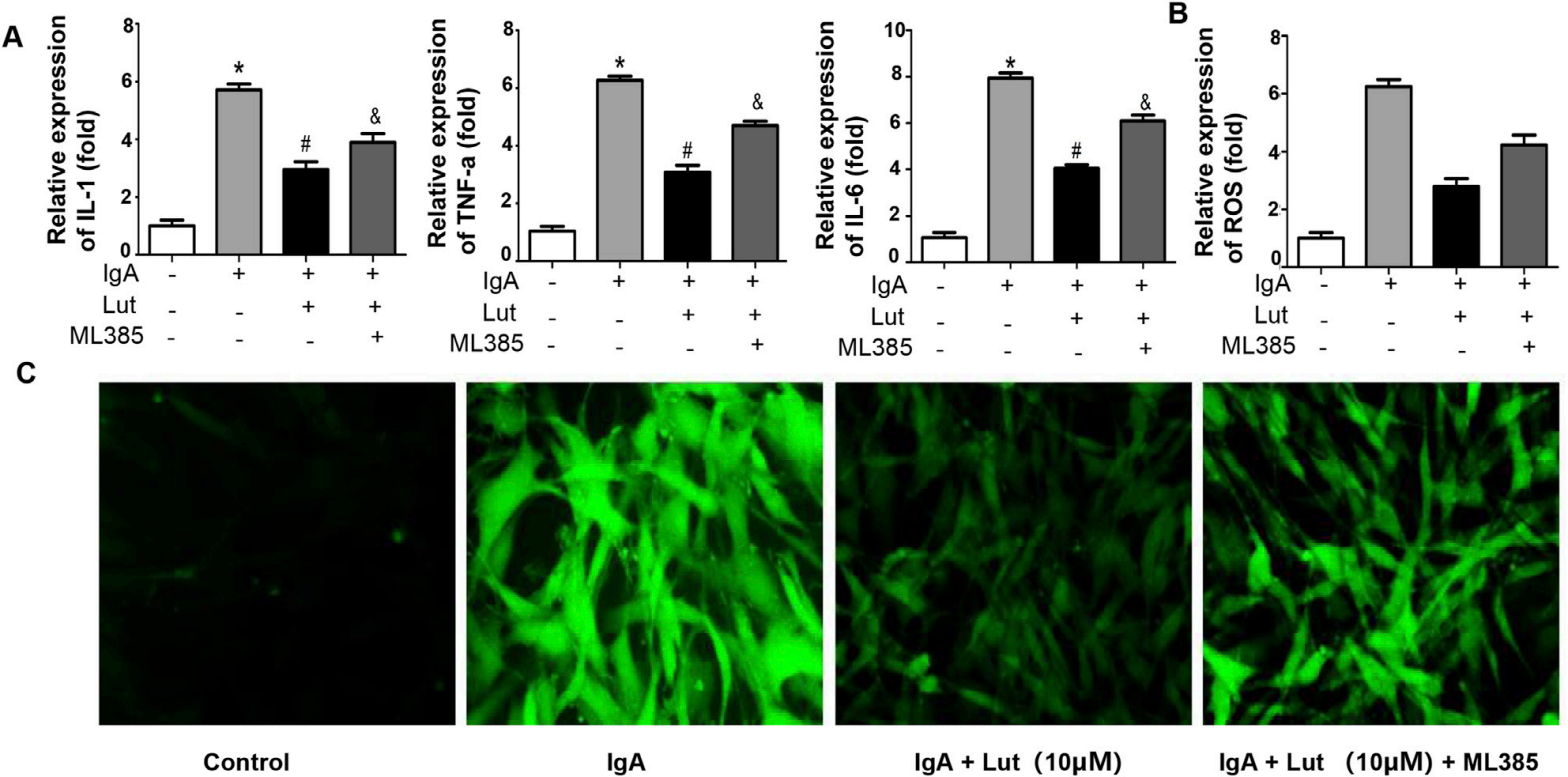
Nrf2 inhibition accelerates Gd-IgA1-stimulated inflammation and oxidative stress**. (A)** Levels of TNF-α, IL-1, and IL-6 were assessed via ELISA. **(B)** ROS levels quantified by ELISA. **(C)** ROS generation detected by DCFH-DA staining (100×). Results presented as means ± standard deviations (n = 3), *p < 0.05 vs the control group; #*p* < 0.05 vs the IgAN group.

**FIGURE 8:**
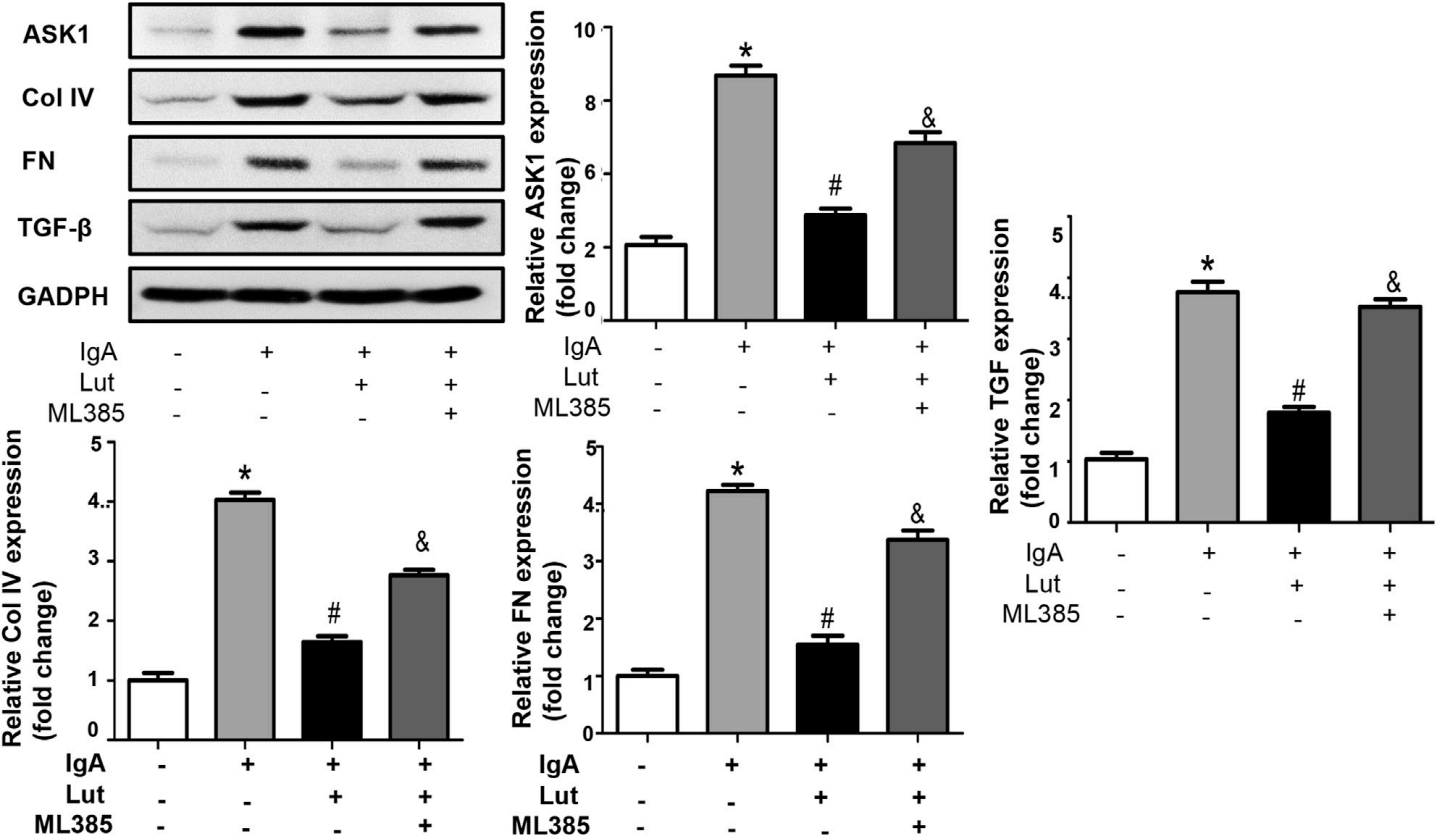
Inhibition of Nrf2 reverses the effects of Lut on ECM production. Protein expression of ASK1, Col IV, FN, and TGF-β was measured by Western blotting and quantified via ImageJ. Results presented as means ± standard deviations (n = 3), *p < 0.05 vs the control group; #*p* < 0.05 vs the IgAN group.

To investigate the impact of Nrf2 on the protection of Lut against Gd-IgA1-induced death in HBZY-1 cells, we applied the Nrf2 inhibitor ML385. As indicated by the ELISA results, the levels of TNF-α, IL-1, and IL-6 were evidently decreased when the Gd-IgA1-induced cells were treated with 10 μM Lut. However, inhibition of Nrf2 expression increased the levels of these proinflammatory factors (Figure 7A). Besides, a reduction in ROS levels was noted in cells treated with Lut in contrast to those exposed to Gd-IgA1, whereas Nrf2 inhibition reversed the inhibitory effect of Lut on the ROS production induced by Gd-IgA1 (Figures 7B,C). In addition, the expression of proteins related to ECM accumulation, including ASK, TGF-β1, and Col IV, was increased due to Nrf2 inactivation (Figure 8). Overall, these results indicate that Lut inhibits the inflammation induced by Gd-IgA1. Furthermore, the activation of Nrf2 leads to a reduction in oxidative stress and a decrease in ECM accumulation.

## Discussion

IgAN is a common chronic kidney disease that is found during a kidney biopsy. Typically, primary IgAN arises from the accumulation of IgA macromolecules within the glomeruli, harming the glomeruli and the interstitial area of the renal tubules. At present, the pathogenesis of IgAN is unclear, and the disease is generally related to the permeability of spherical filter membranes. It is believed that oxidative stress and the secretion of various inflammatory factors are risk factors for IgAN onset (Stamellou et al., 2023).

Lut is a flavonoid compound that plays a role in anti-inflammatory processes and affects other intracellular activities, such as proliferation, metastasis, and antioxidant activity. Rath et al. (2024) demonstrated the inhibitory effects of Lut on cell growth, migration, and epithelial‒mesenchymal transition (EMT) in hepatoma cells. Deshmukh et al. (2024) demonstrated that Lut enhances the function of mitochondria in db/db mice by inhibiting oxidative stress and inflammation. In the present study, a mouse model of IgAN was established, leading to elevated levels of urine ACR and renal function indicators. The Lut treatment group received Lut treatment for 8 weeks. Significant improvements were observed in the urine ACR and renal function indices of the mice, along with amelioration of kidney pathological changes. These findings suggest that administering Lut notably reduces renal injury in IgAN mice.

Several studies have indicated that the activation of ROS within the kidney is a significant factor in the pathophysiology of IgAN patients and experimental models of the disease (Ohashi et al., 2009). The induction of oxidative stress pathways in human mesangial cells can occur through *in vitro* stimulation with Gd-IgA1 (Coppo et al., 2010). Indicators of oxidative stress, including elevated levels of the lipid peroxide MDA, have been identified in the kidney tissues of IgAN patients (Zhang et al., 2025). Similar findings have been observed in the serum and red blood cells of these patients (Krata et al., 2021). In addition, elevated markers of oxidative stress have been reported to be associated with proteinuria and disease progression in IgAN patients (Caliskan et al., 2022). In our study, there was a significant increase in ROS and MDA levels, accompanied by a notable decrease in GSH levels, in the kidney tissue of mice in the IgAN model group, suggesting the significant involvement of ROS in the progression of kidney damage in IgAN mice. Following the administration of Lut, there was a significant reduction in the ROS and MDA levels in renal tissue, suggesting a potential protective effect of Lut on kidney injury in IgAN via a decrease in ROS. In the *in vitro* experiments, Lut inhibited the Gd-IgA1-induced proliferation of HBZY-1 cells. Compared with those in IgA-treated cells, the production of TNF-α, IL-1, and IL-6 was suppressed, and ROS levels were decreased in Lut-treated cells, suggesting the inhibitory effects of Lut on Gd-IgA1-stimulated rat glomerular mesangial cell proliferation, inflammation, and oxidative stress.

Kidney fibrosis is a common pathway involved in the progression of all chronic kidney diseases. ECM accumulation contributes to glomerular fibrosis by thickening the basement membrane of the glomerulus and expanding the mesangium. Inflammation results in the abnormal stimulation of renal cells such that excessive TGF-β1 generation is promoted; the progressive deposition of extracellular matrix proteins, including FN, LN, and collagen proteins, is induced, leading to metabolic abnormalities in mesangial cells (Theocharis et al., 2016; Li et al., 2022). In this study, Masson staining revealed a significant increase in the renal fibrosis area in IgAN mice compared with that in control mice, which was alleviated by the administration of Lut. Subsequent Western blotting analysis demonstrated that Lut effectively impeded the overproduction of extracellular matrix proteins such as Col IV and FN. Moreover, the levels of serum proteins and the expression of mRNAs related to fibrosis markers, such as Col IV, FN, and LN, were also reduced in the Lut treatment group. In general, Lut alleviated fibrosis progression in IgAN, as evidenced by the cell experiment results. Treatment with Gd-IgA1 significantly enhanced the release of TGF-β1, FN, LN, and collagen types in mesangial cells. However, Lut reversed the induction effect of Gd-IgA1 on the extracellular matrix protein HBZY-1.

The Nrf2/HO-1 pathway plays a role in modulating inflammatory reactions through diverse signaling pathways. Studies have demonstrated the pivotal role of Nrf2 in orchestrating anti-inflammatory and antioxidant responses (Kobayashi et al., 2016). The up-regulation of Nrf2 expression triggers the activation of HO-1, leading to the resolution of inflammation (Yuan et al., 2018). Research has highlighted the effectiveness of Lut in mitigating acute lung injury induced by lipopolysaccharides in murine models and alleviating endometritis triggered by LPS through the activation of the Nrf2 signaling pathway (Xiong et al., 2024; Shaukat et al., 2024). Moreover, by activating the Nrf2-mediated signaling pathway, Lut effectively inhibits the inflammatory response and apoptosis of renal tissue and cells (Albarakati et al., 2020). This study revealed increases in the expression levels of Nrf2, HO-1, and NQO1 upon Lut administration in IgAN renal tissues and Gd-IgA1-induced HBZY-1 cells, suggesting the involvement of the Nrf2/HO-1 pathway in protecting the kidneys against Gd-IgA1-induced injury. Further investigations revealed that Nrf2 inhibition resulted in the suppression of Nrf2, HO-1, and NQO1 activation in the presence of Lut, leading to the reversal of ECM-related protein expression. Furthermore, a significant reversal of ROS, MDA, GSH, and inflammation was observed. The inhibition of Nrf2 generally counteracts the inhibitory effect of Lut on fibrosis and its protective effect on IgAN.

However, the mechanism of fibrosis development in IgAN is very complex, and our study has several limitations. First, Lut was administered orally. Although all the animals were alive at the end of the experiment, side effects may have occurred in other tissues. Second, we explored only the effects of Lut on inflammation, oxidative stress, and ECM accumulation in HBZY-1 cells, and we reported the role of the Nrf-2/HO-1 signaling pathway in Lut attenuated inflammation, oxidative stress, and ECM accumulation. The relationship between oxidative stress and ECM accumulation was not further explored.

We found in this study that Lut, which acts as an antioxidant, can prevent glomerular damage induced by IgA by blocking inflammation and oxidative stress and decreasing extracellular matrix buildup. This effect may be achieved by activating the Nrf2/HO-1 signaling pathway, which positions Lut as a promising candidate for treating IgAN. However, the specific molecular targets of Lut and whether and to what extent it also exerts effects *in vivo* require further research.

## Data Availability

The original contributions presented in the study are included in the article/supplementary material; further inquiries can be directed to the corresponding author.

## Abbreviations

ARE, activating antioxidant response element; ACR, albumin/creatinine ratio; BUN, blood urea nitrogen; Col IV, collagen 4; DMEM, Dulbecco’s modified Eagle’s medium; ESRD, end-stage renal disease; ECM, extracellular matrix; EMT, epithelial‒mesenchymal transition; FBS, fetal bovine serum; FN, fibronectin; GSH, glutathione; HO-1, heme oxygenase-1; HE, hematoxylin‒eosin; IgAN, IgA nephropathy; IL-6, interleukin 6; Lut, luteolin; LPS, lipopolysaccharide; LN, laminin; MDA, malondialdehyde; Nrf2, nuclear factor erythroid derived 2-related factor 2; OD, optical density; PFA, paraformaldehyde; PAS, Periodic Acid-Schiff; ROS, reactive oxygen species; TNF-α, tumor necrosis factor-α; TGF-β, growth factor-β.
